# Multiclass Informative Instance Transfer Learning Framework for Motor Imagery-Based Brain-Computer Interface

**DOI:** 10.1155/2018/6323414

**Published:** 2018-02-22

**Authors:** Ibrahim Hossain, Abbas Khosravi, Imali Hettiarachchi, Saeid Nahavandi

**Affiliations:** Institute for Intelligent Systems Research and Innovation, Deakin University, Geelong, VIC, Australia

## Abstract

A widely discussed paradigm for brain-computer interface (BCI) is the motor imagery task using noninvasive electroencephalography (EEG) modality. It often requires long training session for collecting a large amount of EEG data which makes user exhausted. One of the approaches to shorten this session is utilizing the instances from past users to train the learner for the novel user. In this work, direct transferring from past users is investigated and applied to multiclass motor imagery BCI. Then, active learning (AL) driven informative instance transfer learning has been attempted for multiclass BCI. Informative instance transfer shows better performance than direct instance transfer which reaches the benchmark using a reduced amount of training data (49% less) in cases of 6 out of 9 subjects. However, none of these methods has superior performance for all subjects in general. To get a generic transfer learning framework for BCI, an optimal ensemble of informative and direct transfer methods is designed and applied. The optimized ensemble outperforms both direct and informative transfer method for all subjects except one in BCI competition IV multiclass motor imagery dataset. It achieves the benchmark performance for 8 out of 9 subjects using average 75% less training data. Thus, the requirement of large training data for the new user is reduced to a significant amount.

## 1. Introduction

Brain-computer interface (BCI) is a system that establishes a communication channel between the brain and control devices without using the neuromuscular system of human body [1].

One of the noninvasive modalities of BCI is electroencephalography (EEG). BCI uses different types of EEG control signal from the external scalp of the brain. Some of the control signals used in BCI are visual evoked potential (VEP), P300 evoked potential, slow cortical potential (SCP), and sensory-motor rhythms (SMR) [2]. SMR can be modulated by actual as well as imagery motor task by user [3, 4]. Thus, SMRs are used in BCI as the control signal for translating motor task (hand and foot movement) [5] and referred to as motor imagery- (MI-) BCI. Hence, MI-BCIs are used for supporting patients with spinal cord injury and stroke [6–8]. MI-based BCI system possesses some drawbacks such as lack of robustness, complex setup, and long calibration time [1, 9, 10].

Generally, it is recommended to use at least five times more training data per class than the features [11, 12]. Channel-frequency-time information makes the feature vector of EEG signal very high-dimensional [13, 14]. These high-dimensional features necessitate the requirement for a large number of EEG epochs to be collected to train the classifier [15, 16]. But, EEG data acquisition is a lengthy and exhaustive task for the user. For motor imagery purpose, it is sometimes a day-long process [10, 16]. EEG signals recorded from the scalp are very subjective. It varies from one subject to another for same tasks. Even, it differs for same subject in different sessions [4]. Consequently, each individual has to go through this long data collection process in each attempt of using the system. It is most likely that long calibration time for a user has become one of the bottlenecks of BCI system. Calibration time reduction approaches reported in literatures also reflect the scenario well [17–22].

If labeled samples for certain tasks are available from other users, these samples can be used for a new user. The objective is to utilize the knowledge from data spaces of past users to learn predictive function for a new user. This process of knowledge and information conveying from other domain is known as transfer learning (TL) [23].

TL has been applied for BCI in two types: domain adaptation and rules or knowledge sharing [24]. Some of the domain adaptation approaches are subject invariant common space [18, 19, 25–28], common stationary subspace transfer [29–31], conditional and marginal distribution adaptation [32, 33], and subject-to-subject adaptation [34]. Rule adaptation or sharing prior learning to learn new user prediction function has been attempted in [20, 26, 28, 35]. Active transfer learning (ATL) approach was proposed and implemented by Wu et al. in [36] for VEP based BCI. In their work, actively learned samples from the domain of new coming subject were combined with the samples of historical subjects. QBC was used as active learning method to select samples from the subject-specific domain. The authors used all samples from other subjects directly without any adaptation or selection. An improved version of ATL was proposed and implemented for binary MI-based BCI in our preliminary work [37, 38]. Both works implemented ATL on binary classification. In [36], authors did it for target and nontarget VEP while our preliminary work was done on left-hand and right-hand motor imagery classification with two different feature extraction processes in sequence. Since instances are transferred directly from the source to target domain, it is named as direct transfer with active learning (DTAL). DTAL needs to be investigated for multiclass BCI. Instead of direct transfer, an informative and functional subdomain transfer from source to target also needs to be introduced in DTAL. In addition to finding actively learned samples from target domain (in DTAL), active learning based on most uncertain samples from the source to target domain is introduced in this work. To serve these purposes, the following attempts are made in this paper:

- Multiclass direct transfer with active learning (mDTAL) is formulated and implemented. It is the multiclass extension of active transfer learning proposed in [36] for motor imagery BCI (Section 3.1).
- Then, aligned instance transfer is introduced for multiclass MI-based BCI (Section 3.2).
- After that, informative instances transfer framework is formulated and implemented with and without aligned subspace. Here, multiclass entropy as uncertainty criterion is applied in the source to target domain transfer (Section 3.3).
- To address the subject-dependent performance variation of different methods, a generic optimized weighted ensemble of all proposed methods is constructed and applied (Section 3.4).

The main goal of this work is to develop an informative transfer learning framework for MI-BCI which is expected to perform better than direct transfer (mDTAL).

The rest of the paper is organized as follows: Section 2 will describe the concept of different terms and methods which are used for further algorithm's development.  Section 3 will describe developed multiclass frameworks and optimized ensemble method.  Section 4 will describe experimental setup. Then, Section 5 will analyze and discuss the results. Finally, Section 6 will conclude the paper with the scope of future improvement.

## 2. Methods

### 2.1. Transfer Learning (TL)

At first, we need to define some terms to state our problem in the scope of transfer learning.

*Domain*. A domain *D* consists of {*χ*, *P*(*X*)}. Here *χ* is features of *n* dimension (*x*_1_, *x*_2_,…, *x*_*n*_) and *P*(*X*) means marginal distribution. So, *D*_*S*_ = *D*_*T*_ means *P*_*S*_(*X*) = *P*_*T*_(*X*) and *χ*_*S*_ = *χ*_*T*_. Similarly, *D*_*S*_ ≠ *D*_*T*_ means *P*_*S*_(*X*) ≠ *P*_*T*_(*X*) or/and *χ*_*S*_ ≠ *χ*_*T*_ [23].

*Task*. *T* = {*Y*, *f*(·)}, where* Y* is set of all class label and *f*(·) is prediction function which is trained on {*X*, *Y*}. From probabilistic view point, *f*(·) will give conditional probability *P*(*Y*∣*X*). So *T*_*S*_ ≠ *T*_*T*_ means *Y*_*S*_ ≠ *Y*_*T*_ or /and *P*_*S*_(*Y*∣*X*) ≠ *P*_*T*_(*Y*∣*X*) [23].

*Transfer Learning [23]*. Given a source domain *D*_*S*_ and learning task *T*_*S*_, a target domain *D*_*T*_, and learning task *T*_*T*_, transfer learning aims to help improve the learning of the target predictive function *f*_*T*_(·) on *D*_*T*_ using the knowledge in *D*_*S*_ and *T*_*S*_, where *D*_*S*_ ≠ *D*_*T*_ or *T*_*S*_ ≠ *T*_*T*_.

Dataset of EEG epochs from a new user is the target domain. EEG epochs with the label from past users are source domain. Same feature extraction method has been applied for both target and source EEG epochs. So, it can be implied that *χ*_*T*_ = *χ*_*S*_. Same types of classes are labeled for imagery EEG epochs in both source and target domain. It implies that *Y*_*T*_ = *Y*_*S*_. But, different subjects neural responses to same motor imagery action have different characteristics. As a result, marginal distribution and conditional distribution are different for source and target domain [33]. That means *P*_*S*_(*X*) ≠ *P*_*T*_(*X*) and *P*_*S*_(*Y*∣*X*) ≠ *P*_*T*_(*Y*∣*X*).

So, samples from source domain cannot represent the target domain correctly. Hence, it needs to get some subdomain from source efficiently which is related mostly to the target domain. The aim of TL is to learn a target prediction function *f*(*X*_*T*_) → *Y*_*T*_ so that expected error on *D*_*T*_ is as low as possible while *P*_*S*_(*X*) ≠ *P*_*T*_(*X*) and *P*_*S*_(*Y*∣*X*) ≠ *P*_*T*_(*Y*∣*X*).

In this paper, our approach is to select the most informative instances from source domains with the help of few samples of the target domain. Then, we will add them to target domain samples to train a classifier for predicting the label of independent test data of target domain.

### 2.2. Active Learning (AL)

**Figure 1:**
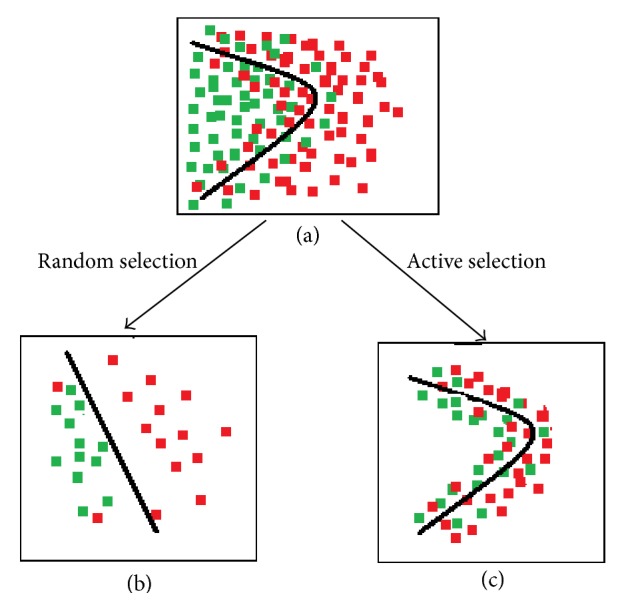
Visual presentation of AL. (a) 2D presentation of binary class dataset with expected decision boundary. (b) Learned decision line on randomly selected samples. (c) Learned decision line on actively selected samples which is more close to expected line.

Active learning method queries for unlabeled samples which have most uncertainty [40]. Trained hypothesis on labeled samples gets confused over some unlabeled samples. These samples are more close to decision line. So, labeling these uncertain samples will accelerate learning process of the model. Hence, these samples carry more information than other certain samples (Figure 1). In this work, active learning method is applied to two ends. At first, query by committee is applied to select the most informative samples from target domain.

Then, entropy is applied as uncertainty measure to select informative samples from the source domain.

**Figure 2:**
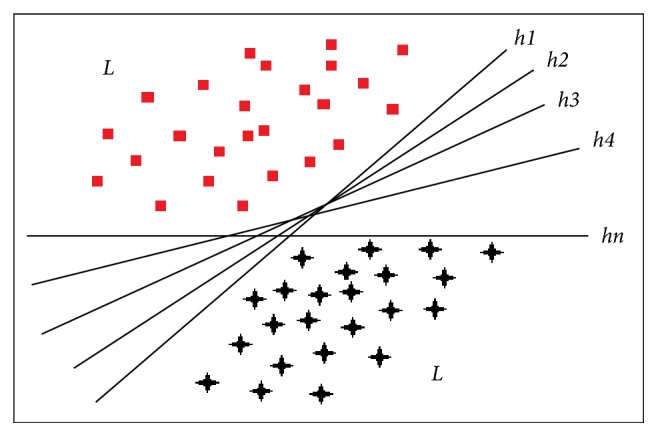
Illustration of linear version space. Each hypothesis in version space is consistent on *L* (labeled training set). Each of them represents different regions of version space.

*Query by Committee (QBC) [41].* A hypothesis is a kind of particular set of parameters that tuned on some training set and it can make the prediction over new data. Hypothesis space is all possible set of hypotheses. Version space is a subset of these hypotheses which are consistent with the labeled training set *L* (Figure 2). Consistent means that the member of version space can make a correct prediction on all instances of *L*. One of the aims of AL is to select instances which can narrow down this version space. It will make the process of learning target prediction function more precise with fewer labeled instances.

QBC maintains a committee of hypothesis (version space) *C* = {*C*_0_, *C*_1_, *C*_2_,…, *C*_*M*_} (Figure 2). Each member of this committee is trained on labeled data *L* and represents a candidate hypothesis (*h*_1_, *h*_2_,…, *h*_*n*_). Then, each member of committee votes for unlabeled samples about their label. The instances attaining the most disagreement about label among the members are considered as the most informative. In analytical perspective, QBC implementation has two steps:

- Construction of committee of hypotheses which depict various regions of version space from specific to general (Figure 2)
- Quantification of disagreement among the members of the committee.

In this work, linear discriminant analysis (LDA) is our learning model. This model gives negative decision score for one class and positive for others. So, decision boundary ideally is zero scoreline. It is unlikely to get extreme negative (−1) at the same time extreme positive (+1) score for a single sample. Certain instances will have the extreme sum of decision score for which most of the members are agreed. But, uncertain instances will not have the extreme score for any class. It makes the absolute value of the sum of the score for all classes close to zero. In case of LDA, ensemble sum of decision score close to zero represents more disagreement among the members. So, instances attaining the lowest absolute value of the algebraic sum of decision scores from members of the committee are the most informative.

*Entropy*. Entropy is the amount of information to encode a distribution [42]. It is used as the measurement of uncertainty. For binary classification, entropy enforces us towards posterior probability 0.5. For multiclass, entropy yields a central confusing area of posterior probability. It considers probability distributions for all classes.

(1) $$\begin{array}{c} x_{Entropy}=\underset{x_{i}}{max}-\overset{n_{C}}{\underset{c=1}{\sum }}P_{\theta }\left(\;y_{c}\;|\;x_{i}\;\right)\log P_{\theta }\left(\;y_{c}\;|\;x_{i}\;\right). \end{array}$$

Here, *P*_*θ*_(*y*_*c*_∣*x*_*i*_) is the predicted probability of *i*th sample *x*_*i*_ for class *y*_*c*_ by the model *θ*. *n*_*C*_ is the number of classes.

### 2.3. Feature Extraction: Common Spatial Pattern (CSP)

This method maximizes the variance for one task and minimizes the variance for other task. Therefore, it yields to generate discriminating features of two classes for EEG classification [43–45]. Let us consider that *X*_*i*_ ∈ *R*^ch×*t*^ is the *i*th single-trial bandpass EEG signal and *Z* ∈ *R*^ch×*t*^ is the spatially filtered signal with CSP projection matrix *A* ∈ *R*^ch×ch^. Here, ch is the number of channels and *t* is the number of time points in single-trial bandpass EEG epoch.

(2) $$\begin{array}{c} Z=A^{T}X_{i}. \end{array}$$

Δ_1_ and Δ_2_ are the covariance matrixes of EEG signals *X* for two classes which can be obtained by

(3) $$\begin{array}{c} \Delta_{Y}=\frac{1}{n_{Y}}\underset{i\in I_{Y}}{\sum }X_{i}X_{i}^{T}\;Y=\left[1,2\right]. \end{array}$$

Here, *I*_*Y*_ is the set of indices of trials corresponding to class *Y* and *n*_*Y*_ is the total number of trials from class *Y*. *A* is the transformation matrix satisfying below optimization.

(4) maxA ATΔ1AATΔ2A.

This CSP filter matrix *A* can be obtained by solving

(5) $$\begin{array}{c} \Delta_{1}A=\left(\;\Delta_{1}+\Delta_{2}\;\right)AD. \end{array}$$

Here, *D* is a diagonal matrix and it contains eigenvalues.

Generally, *m* first and *m* last rows of *A* (represented by *A*^*∗*^ ∈ *R*^*c*×2*m*^) make the spatial filtered signal *Z*^*∗*^ [46]:

(6) $$\begin{array}{c} Z^{*}=X^{T}A^{*}. \end{array}$$

Finally, logarithm of variance of *Z* will give the feature vector *F*.

(7) $$\begin{array}{c} F=\log\left(\;\mathrm{var}\left(\;Z^{*}\;\right)\;\right). \end{array}$$

This CSP is for binary class. We have used four *one*  *vs*  *rest* binary CSP for four classes implementation [44].

### 2.4. Linear Discriminant Analysis (LDA)

LDA is simple and fast to compute [47, 48] which is very successfully paired with CSP feature extraction for MI-based BCI. For binary classification, it deals with two scatter matrixes *S*_*w*_ and *S*_*b*_ which are named as* within-class* and* between-class* scatter. *S*_*w*_ and *S*_*b*_ are defined as follows:

(8) Sw=1n∑i=1k∑j=1nixij−ωixij−ωiT

(9) Sb=1n∑i=1kniωi−ωωi−ωT.

Here, *ω*_*i*_ denotes the mean vector of *i*th class and *ω* denotes the total mean vector. *k* and *n* are number of classes and total number of samples, respectively. Objective is set to find matrix *G* for transformation such that it can ensure maximization of between-class and minimization of within-class scatter. In this work, four *one*  *vs*  *rest* LDA classifiers are used for 4-class classification.

(10) maxG trGTSbGtrGTSwG.

Decision score is calculated by

(11) $$\begin{array}{c} f\left(\;x\;\right)=Gx+b. \end{array}$$

Here, *b* is the bias value and sign of *f*(*x*) will give the class label.

## 3. Algorithms

### 3.1. Multiclass Direct Transfer with Active Learning (mDTAL)

**Figure 3:**
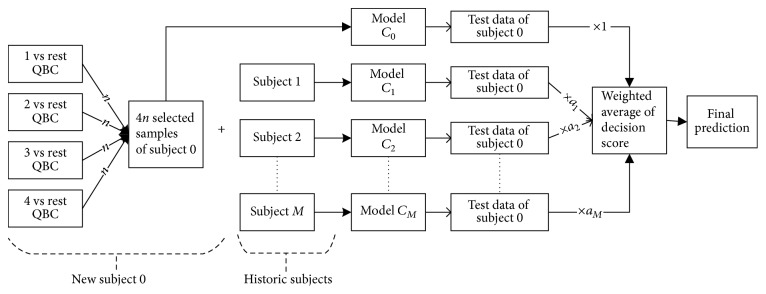
Multiclass direct transfer with active learning (mDTAL). Here, *n* is the number of samples to be selected from each class of new subject.

Multiclass extension of direct transfer learning with active learning or ATL [36] is formulated for MI-based BCI. CSP is used for feature extraction combined with LDA classifier since this combination is very successful for MI-based BCI [16, 46]. For mDTAL, we have considered *one*  *vs*  *rest* approach [49, 50] in three sections of this algorithm (Figure 3):

- *One*  *vs*  *rest* method for QBC while selecting most active samples from target domain
- *One*  *vs*  *rest* CSP filter in feature extraction part
- *One*  *vs*  *rest* method for LDA training and testing part.

Stepwise process of mDTAL algorithm is described as follows.

*Algorithm: mDTAL*

**Step 1 .**

Start with randomly chosen *N*^*l*^ labeled samples with equal class proportion and *N*^*u*^ unlabeled instances from target domain. *M* number of other subjects with *N*^*m*^ labeled instances from *m*th subject are available. *N*^*t*^ independent test samples of new subject are given for performance evaluation.

**Step 2 .**

Train classifier *C*_0_ using *N*^*l*^ samples. Then, *C*_0_ will calculate the decision score for each class of *N*^*u*^ samples as *D*_*u*_^0^.

**Step 3 .**

Train combined classifier *C*_*m*_ using *N*^*l*^ ∪ *N*^*m*^ combined training samples.

**Step 4 .**

Get 10-fold cross-validation accuracy *a*_*m*_ on *N*^*l*^ ∪ *N*^*m*^ training samples. Repeat Steps 2 and 3 for all historic subjects.

**Step 5 .**

Get ensemble weighted average decision score for each class on *N*^*u*^ using the following equation:

(12) $$\begin{array}{c} D_{u}=\frac{D_{u}^{0}+\sum_{m=1}^{M}\left(a_{m}*D_{u}^{m}\right)}{1+\sum_{m=1}^{M}a_{m}}. \end{array}$$

Here, *D*_*u*_^*m*^ is decision score calculated using classifier *C*_*m*_ on unlabeled samples *N*^*u*^. For *D*_*u*_^0^, weight has been assigned as 1 to give subject-specific classifier higher priority over combined classifier. Similarly, ensemble weighted average decision score for test data set *N*^*t*^ is also calculated as follows:

(13) $$\begin{array}{c} D_{t}=\frac{D_{t}^{0}+\sum_{m=1}^{M}\left(a_{m}*D_{t}^{m}\right)}{1+\sum_{m=1}^{M}a_{m}}. \end{array}$$

It is the ultimate output of the algorithm in each iteration.

**Step 6 .**

Linear classifier LDA has the negative score for one class and positive for other. So, decision score close to zero represents more uncertainty than others. Equation (12) calculates ensemble decision score of the *M* + 1 number of models or a committee of models. Unlabeled samples getting lowest or close to zero absolute decision score are more likely to learn decision boundary than others. Considering multiclass, *D*_*u*_(:, *c*) gives decision score for class *c*  *vs*  *rest*. So, the lowest absolute decision score of *D*_*u*_(:, *c*) will give most uncertain samples near class *c*  *vs*  *rest* boundary as follows (Figure 6):

(14) $$\begin{array}{c} S_{c}=\underset{n}{min}\;ascend\left\{\;\text{abs}\left\{\;D_{u}\left(\;\text{:},c\;\right)\;\right\}\;\right\}. \end{array}$$

Here, *c* = 1,2,…, *n*_*C*_ (number of class) and *n* is number of samples to be selected from each class (Figure 3).

**Step 7 .**

All selected unlabeled subject-specific samples *S*_*c*_ are queried for label. Then this newly labeled samples are added to *N*^*l*^ and removed from *N*^*u*^. Steps 2to 7 are repeated until maximum number of iteration.

### 3.2. Multiclass Aligned Instance Transfer with Active Learning (AITAL)

**Figure 4:**
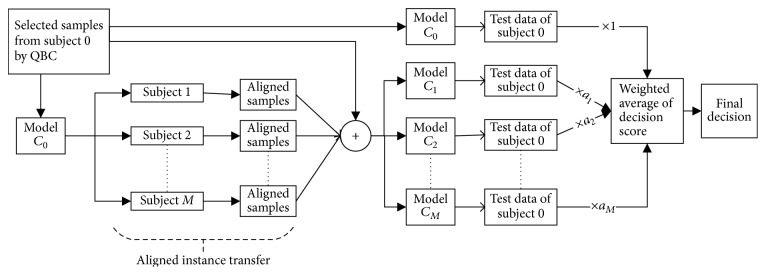
Multiclass aligned instance transfer with active learning (AITAL).

There is no adaptation or selection from historic subjects in mDTAL method. Rather, it directly transfers all labeled samples from historic subjects. But, all samples from historic subjects may not be compatible with the domain of new subject. As a result, it may yield to negative transfer effect [51]. So, the idea is to transfer samples which are aligned with new subject decision boundary (Figure 4). Subject-specific model *C*_0_ classifies some samples accurately from historic subjects. It can be assumed that these accurately classified samples agree with the decision boundary of target domain classifier *C*_0_. So, these samples are considered as being aligned with target domain.

AITAL is similar to mDTAL algorithm except Step 3 where it will not take all of *N*^*m*^ samples from *m*th historic subject. Instead, it will take *N*^*m*′^ aligned samples (see (15)) from *m*th historic subject which are determined by subject-specific model *C*_0_ (Figure 4).

(15) $$\begin{array}{c} N^{m^{\prime }}=N^{m}\;|\;\left\{\;L_{0}^{m}==Y\;\right\},\;Y=\left[1,\ldots ,4\right]. \end{array}$$

Here, *L*_0_^*m*^ is the label for samples from *m*th historic subjects which are predicted by subject-specific classifier *C*_0_. *Y* is the true class label for these samples.

### 3.3. Most Informative Instance Transfer with Active Learning (MIITAL)

According to active learning query method, samples lying close to decision boundary are more likely uncertain to be predicted. It makes uncertain samples more informative to learn decision boundary than that of other samples. If informative samples from historic subjects are transferred to learn classifier for new user, it will be more effective. In this work, entropy of instances are used as the quantification of information carried by these samples. Entropy can be calculated by

(16) Ei=−∑cPθyc ∣ xilog⁡Pθyc ∣ xii=1,2,…,Nm,  c=1,…,4.

Here, *P*_*θ*_(*y*_*c*_∣*x*_*i*_) is probability of samples *x*_*i*_ to be in class *y*_*c*_ which is determined by classifier *C*_0_ and represented as model *θ*.

For this work, we consider four *one*  *vs*  *rest* entropy calculation. Our goal is to find uncertain samples which are close to each *one*  *vs*  *rest* decision line. Ideally, samples having 50 : 50 probability ratio are most uncertain and have maximum entropy. We consider samples with probability ratio equal or more than 60 : 40 for this work. It yields to transfer samples that have entropy equal or greater than 0.29228 according to (16). This entropy limit is named information limit or cut-off (*l*).

**Figure 5:**
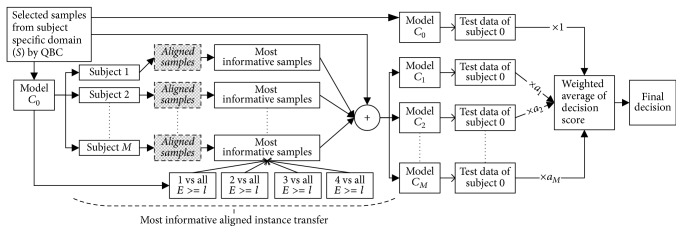
Multiclass most informative and aligned informative instance transfer with active learning (MIAITAL). Shaded box is obsolete for MIITAL.

**Figure 6:**
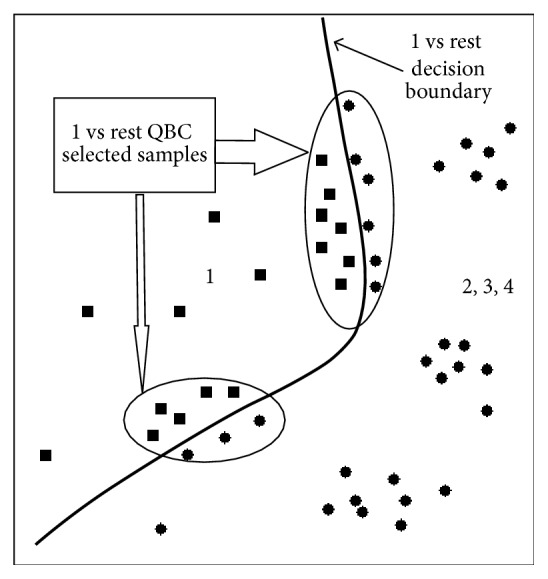
One versus rest presentation of QBC for multiclass.

There are two combinations of this algorithm:

(i) Transfer aligned and most informative samples (most informative and aligned instances transfer with AL (MIAITAL)) (Figure 5):
  (17) $$\begin{array}{c} N^{m^{\prime }}=N^{m}\;|\;\left\{\;L_{0}^{m}==Y,\;\;E\geq l\;\right\},\;Y=\left[1,\ldots ,4\right]. \end{array}$$
(ii) Transfer most informative samples and ignore whether it is aligned or not (most informative instances transfer with AL (MIITAL) (Figure 5)):
  (18) $$\begin{array}{c} N^{m^{\prime }}=N^{m}\;|\;\left\{\;E\geq l\;\right\}. \end{array}$$

Algorithm of MIAITAL or MIITAL is the same as mDTAL except Step 3. In Step 3, MIAITAL or MIITAL will take *N*^*m*′^ according to (17) and (18) in place of *N*^*m*^.

MIAITAL attempts to transfer most informative samples which are perfectly classified by classifier from previous iteration, whereas MIITAL attempts to transfer most informative samples (determined by entropy) and ignores alignment of those informative samples (Figure 5).

### 3.4. Optimized Ensemble for Multiclass Actively Learned Space Transfer

**Figure 7:**
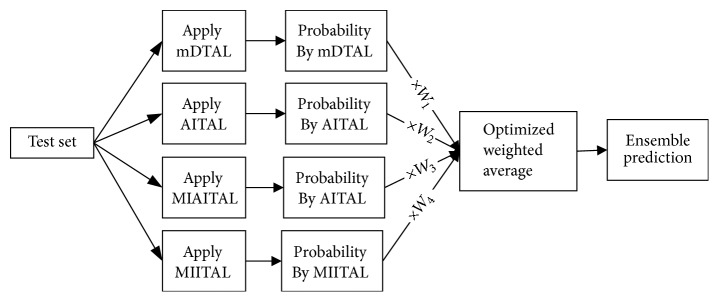
Optimized weighted ensemble of mDTAL, AITAL, MIAITAL, and MIITAL.

EEG epochs due to various motor imagery actions are not stable. So, finding prominent features followed by learning classifier does not always yield the expected result. As a result, performance is not generic for all subjects; it is subject-dependent. Some methods perform well for some subjects while not very good for others. The ensemble of different methods can give a general and steady performance for all subjects. An optimized weighted ensemble is proposed to serve the purpose (Figure 7).

Some arbitrary weight (say *W*) is assigned for each class in each method. Then, these weights are optimized for minimum loss on a validation set. Loss function for *n*th subject is as follows:

(19) $$\begin{array}{c} LOSS_{n}=\underset{i\in V}{\sum }{\left(\;Y_{n}^{t}\left(\;i\;\right)-W_{m}^{c}*P_{m}^{c}\left(\;i\;\right)\;\right)}^{2}. \end{array}$$

Here, the validation set *V* is 10 percent of the subject-specific training set and is randomly chosen from that data set. The initial value of weight *W* is some random value in the range of [0,1]. Then, *W* is optimized by the genetic algorithm using the loss function from (19). Ensemble decision-making probability on test set *T* using optimized *W* is then obtained by

(20) $$\begin{array}{c} P_{opt}^{c}\left(\;x\;\right)=\frac{\sum_{m=1}^{M}W_{m}^{c}*P_{m}^{c}\left(x\right)}{\sum_{m=1}^{M}W_{m}^{c}},\;x\in T. \end{array}$$

Here, *P*_*m*_^*c*^ is probability generated by *m*th method for class *c* and *W*_*m*_^*c*^ is optimized weight for corresponding class *c* and method *m*.

## 4. Experiment Setup

### 4.1. Experimental Data

Algorithms described in Section 3 are implemented for BCI competition IV dataset 2A [39]. This dataset consists of 9 subjects. In this dataset, each subject performs four types of motor imagery action for left hand, right hand, both feet, and tongue movement. Data is recorded in two sessions for each subject. In each session, a subject performs 72 trials per class which turns into 288 in total.

**Figure 8:**
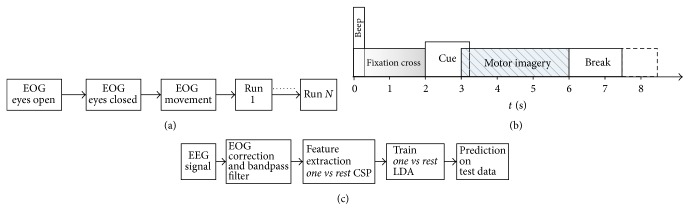
(a) Timing for one session [39]. (b) Timing for a single trial [39]. (c) Baseline method.

In each session, there are approximately 5 minutes of electrooculogram (EOG) recording keeping eyes open, close, and moving. Then, it is followed by the run of trials (Figure 8(a)). Each subject was facing a computer screen which was showing different indication guideline to the subject. Each single-trial starts (*t* = 0 s) with a fixation cross on the screen in front of the subject. After 2 seconds (*t* = 2 s), a cue appeared on the screen indicating arrow with the desired movement sign (left hand, right hand, foot, and tongue). After 1.25 seconds of cue appearing, subject starts to imagine the motor action and continues until *t* = 6 s. A short break (black screen) is given until next trial starts (Figure 8(b)). EEG epoch of 2 seconds after 0.5 seconds of cue appearing is taken as training data. 22 channels (Ag/AgCl) are used for EEG signal recording and other three monopolar electrodes are used for EOG recording. The montage of the electrode was according to the international 10-20 system. Both of the EEG and EOG channels were sampled 250 Hz. After that, they had been filtered using 0.5 Hz to 100 Hz ranged bandpass filter. A 50 Hz notch filter was also enabled during recording to omit the line noise. The sensitivity of the amplifier was set to 100 uV and 1 mV for EEG and EOG recording, respectively.

### 4.2. Data Preprocessing and EOG Correction

Linear EOG correction method [52] is applied for artefact correction on raw EEG signals. *β* rhythms (12–30 Hz) of EEG signals are desynchronized with real movement or motor imagery [53]. *μ* rhythms (8–12 Hz) of EEG signals related to motor actions and sometimes correlate with *β* rhythms [54, 55]. For this reason, corrected EEG signal is bandpass filtered using casual Chebyshev Type II filter between 8 Hz and 32 Hz. After that, CSP is applied and features are extracted according to (6) and (7). Here, *m* is set to 2 for *A*^*∗*^ in (6). So, 4 features are obtained from each EEG epoch.

### 4.3. Experiment and Simulation

For all method, first session of each subject is used as training set and second session is used as test set.

For comparison purpose, a baseline method is also implemented. In baseline method, the full training set of the respective subject is used to train LDA classifier. No sample from other users is used. After applying data preprocessing as described in Section 4.2, four *one*  *vs*  *rest* LDA classifiers are trained. These models are applied to predict label for respective independent test session (Figure 8(c)).

The accuracy achieved by this baseline method is the benchmark performance by an individual user. The purpose of other methods in this work is to achieve this performance using a reduced amount of training samples. This baseline process is followed for each internal model training and testing phase of other algorithms. As benchmark performance is a static value and does not depend on the iterative increment of subjective training samples, it is a straight line parallel to the horizontal axis.

Other methods in this work are iterative where samples from the new subject are added in training pool iteratively. Each subject is considered as the new user (target) while other 8 subjects are considered to be past users (source). Each simulation starts with 40 random samples (*N*^*l*^ in Step 1 of mDTAL algorithm) with equal class distribution from the target domain. Then, 2 samples per class (*n* in (14)) are added in each iteration until 20 iterations (maximum number of iteration in Step 7 of Section 3). So, maximum 200 subjective samples for each subject is added at final iteration. This amount of training samples from the new user is good enough to observe whether the new subject can reach the benchmark using a lower amount of training samples. For this reason, the maximum number of iterations is set to 20. This whole simulation is repeated 20 times for each subject to negate random starting samples effect. Then, the average of ten repeats in each iteration is taken as the performance of that iteration.

Only first session of each historical subjects is taken as source domain because label for the second session was kept closed in BCI competition IV. Training samples from the first session of target subject are added iteratively and the classification performance in each iteration is computed on the independent second session of the target subject.

## 5. Results and Discussion

**Figure 9:**
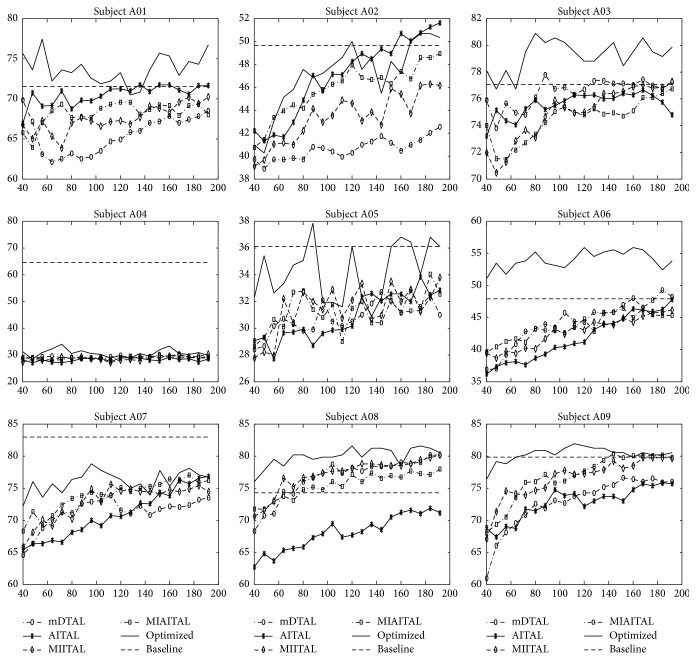
Performance of mDTAL, AITAL, MIITAL, MIAITAL, and optimized ensemble method on BCI competition IV dataset 2A. Accuracy is along the *y*-axis and the number of subject-specific training samples is along the *x*-axis.

The performance of proposed methods in this work is evaluated based on the following two criteria: first, investigation to find whether the method has reached the maximum baseline performance; second, the number of subjects for which intended method reaches the maximum baseline performance. Direct transfer method (mDTAL) is the multiclass extension of active transfer learning [36] for motor imagery BCI. Proposed informative space transfer algorithms (AITAL, MIAITAL, and MIITAL) will be compared with mDTAL based on the evaluation criteria mentioned above.  Figure 9 presents the accuracy of all methods for comparison. The following observations can be drawn out from this result based on the above-mentioned evaluation criteria:

- mDTAL method fails to achieve the baseline performance except for subject A03, A06, and A08. But, it is showing gradual increment in accuracy as the training data from target domain increased. In mDTAL method, all samples from source domain are transferred to the target domain directly. The results reflect that, due to high variability among subjects, there is a high chance of completely different types of domain transfer in direct transfer method.
- AITAL method reaches the baseline for subjects A01, A02, A03, and A06. It depicts that transferring solely aligned information does not always yield to transfer of discriminative features transformation. Widely sparse distribution may be aligned but might not have much information for target domain learning process. Moreover, aligned samples are not ensured to be equal in class distribution. So, there is a high possibility of introducing class-imbalance into the combined domain (source + target).
- MIITAL and MIAITAL transfer most informative samples in each iteration. Both of them reach baseline or close to baseline for subjects A01, A02, A03, A06, A08, and A09. MIITAL shows better performance than MIAITAL. The reason behind this is that MIITAL emphasizes only on information carried out by samples while MIAITAL requires both informative and aligned samples. Some of the informative samples may not be aligned. These nonaligned informative samples with higher entropy are excluded in MIAITAL but are included in MIITAL. Thus, MIITAL outperforms MIAITAL with more informative samples.

From above observation, it is clear that informative transfer approaches (MIITAL and MIAITAL) have reached the baseline for six out of nine subjects while direct transfer (mDTAL) reaches baseline only for three out of nine subjects. It implies that informative subspace transferring enables the new subject to achieve the baseline performance with a reduced number of training data for more number of subjects compared with direct transfer methods.  Table 1 shows the number of subject-specific training samples required to reach baseline or close to baseline. It also implies that MIITAL method reaches baseline or close to baseline using average 49% less subject-specific data for 6 out of 9 subjects.

Though informative instance transfer achieves better performance for most of the subjects, this is not a generic outcome for all subjects. Subject A05 and subject A07 are much closer to baseline, but they do not reach it. Exceptionally, subject A04 is very far from the expected line for all the methods. To find a generic solution for all subjects, an optimized weighted ensemble of the proposed four methods is applied (Figure 7). Performance of optimized weighted ensemble method is shown in Figure 9 (solid black line).

Optimized ensemble of all methods achieves the baseline and sometimes better than baseline with less amount of subject-specific samples for 8 out of 9 subjects. As per results in Table 1, optimized ensemble method reaches the baseline or close to baseline using average 75.5% less subject-specific training samples for 8 out of 9 subjects.

**Figure 10:**
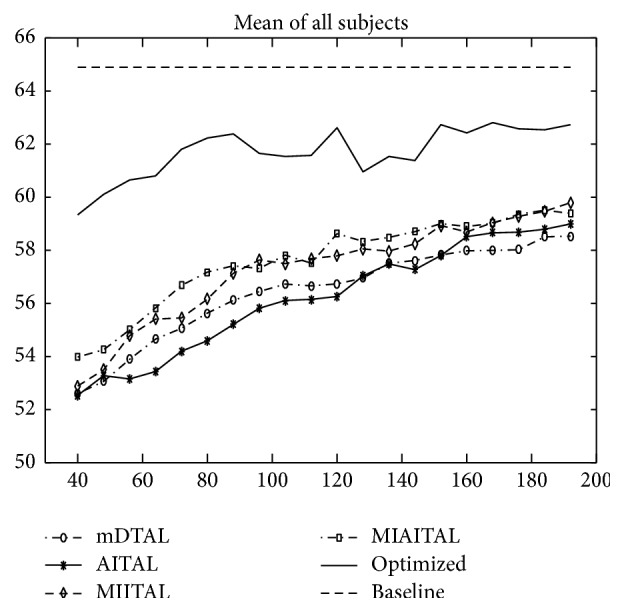
Mean performance of all subjects for mDTAL, AITAL, MIITAL, MIAITAL, and optimized ensemble methods. Accuracy is along the *y*-axis and number of training samples added from the new subject is along the *x*-axis.

To get a generic view irrespective of subjects, mean of the accuracy of all subjects is presented in Figure 10. It shows that proposed informative transfer learning methods MIITAL and MIAITAL are performing always better than that of direct transfer (mDTAL). It infers that informative transfer has advantages over the direct transfer. However, mean performance of both algorithms is behind the baseline performance by 4-5%. On the other hand, mean of the optimized ensemble is much closer to mean baseline of all subjects (differs by only 1-2%). Subjective combination adaptation would have yielded better results in comparison to the optimized ensemble of the methods. However, this will be considered in a future study.

Another observation is that subject A04 has no improvement by all these methods. Any of the methods used in this study is unable to improve the performance of subject A04. This can be due to the fact that EEG response of some subjects have complete dissimilarity with others [55]. When a completely dissimilar subspace is transferred and combined with the target subject (A04), it does not much effect towards the improvement of predictive function for the target domain. A remedy for this issue could be achieved by clustering closely related subject [28]. Closely related subjects or domains form a cluster. Nonrelated or dissimilar subjects are excluded from this cluster. Then, informative subspace from this close group or cluster can make the transfer more effective. For EEG epochs consisting large number of channels, EEG channels selection could be a better addition for robustification [56–60].

Presented results infer that a single method is working well for some subjects and not up to the mark for others. It implies that performance of proposed TL methods is subject-dependent. Automatic selection of the best approach for a subject is an open question to be investigated. One of the possible causes behind the performance variation is CSP applied for extracting features from a broad range of *μ* and *β* rhythms (8–32 Hz). Subjective frequency ranges can be yielded into better feature extraction and selection [49]. Incorporating this subject adaptive frequency ranges will ensure feature transfer from subjective range. Thus, it will lead to better features transfer into proposed TL algorithm. One concerning matter is the mean baseline performance of multiclass BCI that is not up to the mark. Advance feature extraction and learning algorithm could be applied to raise up this baseline which leads to subsequent incorporation into MIITAL and consequent performance raise of the MIITAL algorithm.

In summary, this paper presents two slightly different informative subspace transfer frameworks (MIAITAL and MIITAL) on multiclass BCI. Though MIITAL has achieved the expected result for a good number of subjects, still it is lagging behind the baseline in general. The optimized ensemble of these methods has overcome the gap. The primary goal of this work is to investigate the functionality of informative subspace transferring over the direct transfer for multiclass BCI. Though it succeeded for most of the subjects, there are many scopes to improve in the proposed framework. Secondary goal is to find comparatively better informative transfer approach. From empirical results, it is clear that MIITAL is serving the purpose better than MIAITAL.

## 6. Conclusion

In this work, we applied direct transfer learning with active learning on multiclass motor imagery BCI. To improve the performance, an informative instances transfer framework is proposed. Its key advantage compared with direct transfer methods is transferring informative instances that narrow down the search spaces more precisely around the decision line. Hence, it reduced training data significantly for most of the subjects (6 out of 9). A generic optimized ensemble of proposed methods is also implemented. It has achieved expected accuracy with fewer subject-specific samples (using average 75% less training samples) for 8 out of 9 subjects. Results achieved in this paper point out some directions for future work as well. Subject adaptive method selection could give a more fine-tuned performance. Cluster base transfer combined with informative transferring could also lead to better performance for the underperforming subject. Another scope is filtering subject and subspace based on distribution similarity. Domain adaptation based on marginal and conditional distribution could introduce more generalize adaptation in the proposed TL framework. All these improvements can reduce the calibration effort remarkably and lead us towards a generic TL framework for BCI application.

## Figures and Tables

**Table 1:** Number of samples to reach baseline for different methods.

Sub	Base	MIITAL	Reduction by MIITAL	Optimized	Reduction by optimized
A01	288	180	37.5%	40	86%
A02	288	180	37.5%	90	68.75%
A03	288	140	51.4%	40	86%
A04	288	×	×	×	×
A05	288	×	×	120	58%
A06	288	170	41%	40	86%
A07	288	×	×	100	65.3%
A08	288	60	79%	40	86%
A09	288	150	48%	70	75.7%

Mean	288		49%		76.56%

^*∗*^×: baseline cannot be reached.
